# From Extraction to Stabilization: Employing a 2^2^ Experimental Design in Developing Nutraceutical-Grade Bixin from *Bixa orellana* L.

**DOI:** 10.3390/foods13111622

**Published:** 2024-05-23

**Authors:** Christine L. Luna-Finkler, Aralí da C. Gomes, Francisco C. A. de Aguiar Júnior, Ester Ribeiro, Raquel de Melo Barbosa, Patricia Severino, Antonello Santini, Eliana B. Souto

**Affiliations:** 1 Department of Antibiotics, Federal University of Pernambuco, Cidade Universitária, Recife 50670-901, PE, Brazil; christine.luna@ufpe.br (C.L.L.-F.); ester.ribeiro@ufpe.br (E.R.); 2 Academic Center of Vitória, Federal University of Pernambuco, R. Alto do Reservatório, s/n, Bela Vista, Vitória de Santo Antão 55608-250, PE, Brazil; arali.gomes92@gmail.com (A.d.C.G.); francisco.amanajas@ufpe.br (F.C.A.d.A.J.); 3 Department of Pharmacy and Pharmaceutical Technology, School of Pharmacy, University of Seville, C/Professor García González, 2, 41012 Seville, Spain; 4 Laboratory of Nanotechnology and Nanomedicine (LNMED), Institute of Technology and Research (ITP), Av. Murilo Dantas, 300, Aracaju 49010-390, PE, Brazil; patricia_severino@itp.org.br; 5 Industrial Biotechnology Program, University of Tiradentes (UNIT), Av. Murilo Dantas, 300, Aracaju 49032-490, PE, Brazil; 6 Department of Pharmacy, University of Napoli Federico II, Via D. Montesano, 49-80131 Napoli, Italy; 7 Laboratory of Pharmaceutical Technology, Faculty of Pharmacy, University of Porto, 4050-313 Porto, Portugal; ebsouto@ff.up.pt

**Keywords:** carotenoid, urucum, *Bixa orellana* L., freeze-drying, stability, foodstuff

## Abstract

Bixin is the main carotenoid found in the outer portion of the seeds of *Bixa orellana* L., commercially known as annatto. This compound is industrially employed in pharmaceutical, cosmetic, and food formulations as a natural dye to replace chemical additives. This study aimed to extract bixin from annatto seeds and obtain encapsulated bixin in a powder form, using freeze-drying encapsulation and maltodextrin as encapsulating agent. Bixin was extracted from annatto seeds employing successive washing with organic solvents, specifically hexane and methanol (1:1 *v*/*v*), followed by ethyl acetate and dichloromethane for subsequent washes, to effectively remove impurities and enhance bixin purity, and subsequent purification by crystallization, reaching 1.5 ± 0.2% yield (or approximately 15 mg of bixin per gram of seeds). Bixin was analyzed spectrophotometrically in different organic solvents (ethanol, isopropyl alcohol, dimethylsulfoxide, chloroform, hexane), and the solvents chosen were chloroform (used to solubilize bixin during microencapsulation) and hexane (used for spectrophotometric determination of bixin). Bixin was encapsulated according to a 2^2^ experimental design to investigate the influence of the concentration of maltodextrin (20 to 40%) and bixin-to-matrix ratio (1:20 to 1:40) on the encapsulation efficiency (EE%) and solubility of the encapsulated powder. Higher encapsulation efficiency was obtained at a maltodextrin concentration of 40% *w*/*v* and a bixin/maltodextrin ratio of 1:20, while higher solubility was observed at a maltodextrin concentration of 20% *w*/*v* for the same bixin/maltodextrin ratio. The encapsulation of this carotenoid by means of freeze-drying is thus recognized as an innovative and promising approach to improve its stability for further processing in pharmaceutical and food applications.

## 1. Introduction

*Bixa orellana* L. is a native plant from Tropical America, commonly found between Guianas and the State of Bahia in Brazil, and is a rich source of annatto seeds. Annatto carotenoids are widely used in the food and pharmaceutical industries [1]. In the food industries, they are used as antioxidants [2], photoprotective agents [3], and as natural dyes [4]. From a medicinal point of view, annatto seeds have expectorant properties, laxative, cardiotonic, hypotensive, antibiotic, anti-inflammatory, and healing activities [5,6].

Bixin is a dicarboxylic cis-apocarotenoid monomethyl ester with a high number of conjugated double bonds. It mostly occurs in annatto seeds (*Bixa orellana* L.) [7], making up at least 80% of the total carotenoids in the seed [8]. It has the molecular formula C_25_H_30_O_4_ and is present in the outer coating of the grains of the plant [9]. It is considered a natural dye responsible for shades ranging from yellow to red. 

**Figure 1:**
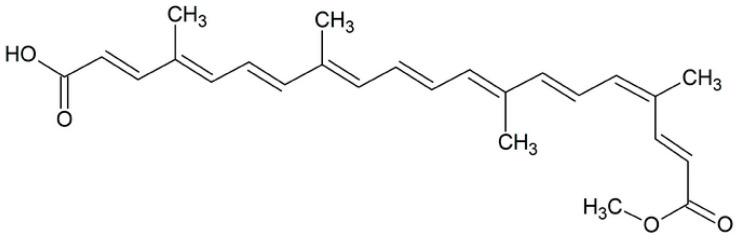
Chemical structure of bixin ((2E,4E,6E,8E,10E,12E,14E,16Z,18E)-20-methoxy-4,8,13,17-tetramethyl-20-oxoicosa-2,4,6,8,10,12,14,16,18-nonaenoic acid, IUPAC nomenclature).

A lengthy chain of polyunsaturated hydrocarbons with methyl ester groups and carboxylic acids at either end makes up the bixin structure (Figure 1). Due to the presence of these functional groups in its chemical structure, the molecule is insoluble in water but soluble in polar organic solvents, such as hexane, methanol, and acetone [10], characterizing it as a fat-soluble compound [11]. The molecule can absorb light at visible spectrum wavelengths, which are close to 440–489 nm, thanks to changes in the chain. This allows for the creation of hues ranging from yellow to orange-red [12]. This ability is due to the presence of a conjugated polyene chain, which has a centrally located double-bond system that acts as a chromophore and is essential for multiple functions and actions [13]. Thus, UV–visible spectroscopy and high-performance liquid chromatography (HPLC) are the most often employed analytical models for the quantification of bixin [9].

The presence of a conjugated polyene chain makes carotenoids prone to isomerization and oxidation [13], making them unstable due to factors such as the presence/absence of oxygen, high temperatures, and light [14].

In recent years, the encapsulation of bioactive natural products has become a critical area of research, particularly for enhancing the stability and bioavailability of these compounds for pharmaceutical and nutraceutical applications [15,16,17]. Encapsulation techniques vary widely but typically involve the use of a matrix or wall material to form a barrier around the active ingredient, thereby protecting it from environmental factors and controlled release [18,19]. Common materials used include lipids, proteins, polysaccharides, and synthetic polymers, each selected based on their biocompatibility, protective properties, and processing characteristics [20,21].

Maltodextrin was selected as the wall material for the encapsulation of bixin, a natural carotenoid derived from *Bixa orellana* L. This choice was informed by maltodextrin’s superior film-forming capabilities, its capacity to act as a protective barrier against oxidation, and its relatively neutral flavor profile, which is especially beneficial in food applications [21]. Furthermore, the high solubility and low viscosity of maltodextrin at elevated concentrations render it particularly suitable for freeze-drying processes, the encapsulation method utilized in this study for bixin [22].

Encapsulation is one method amongst potential alternatives utilized to increase the stability of annatto carotenoids [23,24]. The use of lyophilization as a microencapsulation technique has the advantage of obtaining products with high solubility and oxidative stability. To this end, the drying of the product of interest is carried out by the addition of an encapsulating agent, generally a polymer, as a wall material. According to the choice of encapsulating agent used in the process, particles of different sizes can be obtained [25].

**Figure 2:**
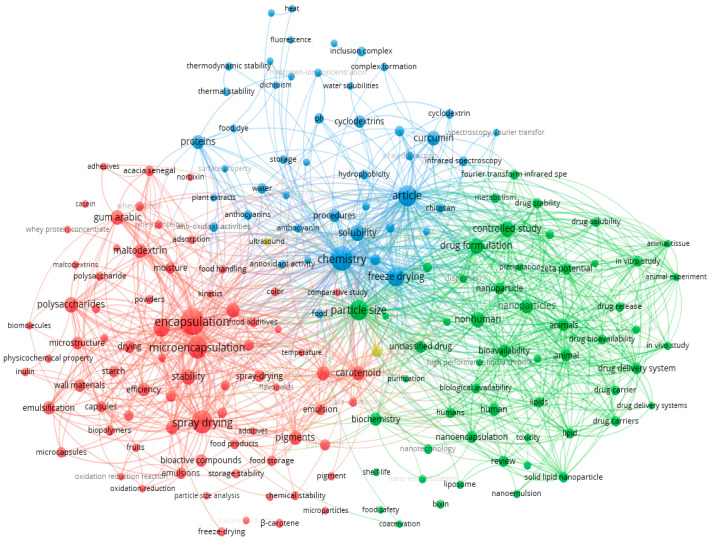
Bibliometric map obtained by VOSviewer software version 1.6.16 (https://www.vosviewer.com, accessed on 25 March 2024), using “bixin” and “freeze-drying” or “lyophilization” as keywords, recorded from Scopus database (recorded on the 25 March 2024).

To document the innovative aspects of this research, we have conducted a survey in Scopus recorded on the 25 March 2024, using a combination of terms, namely, “bixin” and “freeze-drying” or “lyophilization”, which resulted in a total of 205 published papers since ever indexed in the Scopus core collection. VOSviewer software was employed for data analysis, generating the bibliometric map shown in Figure 2 [26]. The outputs were four clusters, covering nano/microencapsulation, animal experimentation, bioactives, and comparative study as the major topics. A total of 117 papers were linked to Agricultural and Biological Sciences, 73 to Chemistry, 54 to Chemical Engineering, 41 to Engineering, 30 to Biochemistry, Genetics, and Molecular Biology, and only 28 were published in the subject area of Pharmacology, Toxicology, and Pharmaceutics which, together with the food sector, is one of the areas of interest for the use of bixin. The novelty of our work relies on the exploitation of strategies to improve the stability of this carotenoid by its encapsulation by freeze-drying, which is for potential use in oral administration (pharmaceutics or foods).

This work aims to encapsulate by lyophilization the carotenoid bixin, using maltodextrin as a wall material. The encapsulation was carried out following a 2^2^ experimental design, having as independent variables the concentration of maltodextrin and the proportion of bixin in relation to the encapsulating material, while the response variables were the encapsulation efficiency and the solubility of the microencapsulated powder.

## 2. Materials and Methods

### 2.1. Materials

Annatto seeds (*Bixa orellana* L.) were purchased from Beleza da Terra company (Space Green FE0444, Curitiba, PR, Brazil), being stored in hermetically sealed polyethylene bags in a refrigerator at 8–10 °C in the Bioprocess Laboratory of the Federal University of Pernambuco, Vitoria Academic Centre (PE, Brazil). The seeds were characterized in terms of protein, lipid, ash, and moisture content, as described below. All used organic solvents with HPLC grade and standard bixin were purchased from Sigma-Aldrich (St. Louis, MO, USA). All other solvents and chemicals were of analytical grade.

### 2.2. Bixin Extraction

**Figure 3:**
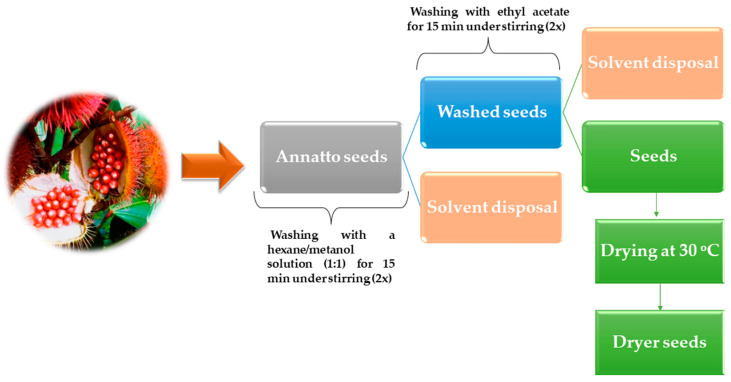
Schematic representation of bixin extraction and washing out of interferences.

Each bixin extraction was performed from 200 g of annatto seeds previously ground and passed through a sieve filter with a size of 48 mesh. Initially, two successive washings of the seeds were carried out with a hexane/methanol solution (1:1) for 15 min under stirring. Then, the seeds were washed twice with ethyl acetate for 15 min under stirring. In this step, the removal of undesirable compounds that affect the purity of the final product occurs, which, in most cases, are composed of lipids and polar compounds. After washing the seeds, they were drained and dried at 30 °C in an oven. The flowchart of the interferents removing process is presented in Figure 3.

After removing the interferents, the seeds were subjected to the extraction process itself in order to obtain the bixin crystals. This step promotes the extraction and purification of annatto extract through crystallization. The dried seeds were washed with dichloromethane and absolute ethanol (in a ratio of 1:4 by volume). Then, the material was taken to an ice bath for 5 min and then frozen. Then, the mixture was filtered, washed with absolute ethanol, and dried at 29 °C in an oven under air circulation for 24 h. The bixin crystals were then kept in an amber flask wrapped in aluminum foil and frozen at −8 °C.

Ten extraction experiments were carried out in order to obtain crystals with a standardized content of bixin and in sufficient quantity to carry out the microencapsulation tests. The bixin content of the extract was evaluated by HPLC. The extraction of bixin crystals has its yield (Y%) calculated by the ratio between the mass of bixin crystals obtained after extraction and the initial mass of seeds (200 g). Thus, the yield was calculated according to Equation (1):

(1) $$\mathrm{Yield}\;(\%)=\frac{\mathrm{mass}\;\mathrm{of}\;\mathrm{cystals}}{\mathrm{mass}\;\mathrm{of}\;\mathrm{seeds}}\times 100\%$$

### 2.3. Spectrophotometric Analysis of Bixin in Different Solvents

For the bixin scanning tests in different solvents, a mass of 25 mg of bixin was added to 30 mL of the solvent under study. After vortexing for 1 min and resting at 30 °C, the samples were centrifuged at 5000 rpm for 5 min. Subsequently, the supernatants were submitted to spectrophotometric analysis in a UV–Vis spectrophotometer (Thermoscientific Genesys 10S, Thermo Fisher Scientific, Waltham, MA, USA) in a wavelength range from 300 to 800 nm. Scanning assays were performed in the following solvents: isopropyl alcohol, ethanol, dimethyl sulfoxide (DMSO), chloroform, and hexane.

### 2.4. Experimental Factorial Design for the Encapsulation of Bixin by Lyophilization

A type 2^2^ experimental statistical design was carried out, with two variables at two levels and quintuplicated at the central point, accounting for a total of nine experiments. The independent variables were the concentration of maltodextrin and the proportion of bixin in relation to the encapsulating material (Mb/Me), while the response variable was the encapsulation efficiency (EE%) and the solubility of the microencapsulated powder. Table 1 shows the values of the variables that were investigated in the experimental design. The complete planning matrix is shown in Table 2. The experiments were performed randomly, and data analysis was performed using the Statistica^®^ program (version 7.0).

Firstly, the mass of maltodextrin indicated by each condition of the experimental statistical design was previously dissolved in water heated to 60 °C to form an aqueous suspension. To facilitate emulsion formation, Tween 80 (0.1%) was added to each maltodextrin aqueous emulsion.

The bixin crystals were dissolved in 5 mL of chloroform, and the mixture was added to the aqueous suspension of maltodextrin. The suspension was vigorously stirred on a magnetic stirrer for 30 min, according to work by Spada et al. (2012) [27], with modifications. Chloroform was chosen (relatively polar solvent, dipole moment 1.04 D) because it has a melting point of −63 °C (1 atm), which allows the samples to be solidified at the temperature used for freezing them (−80 °C) before the lyophilization procedure.

Furthermore, despite being a relatively polar solvent, it is able to solubilize bixin crystals. The samples were distributed in penicillin-type glass vials with a rubber stopper for lyophilization and later frozen at −80 °C overnight. Then, the samples were lyophilized using a lyophilizer (FTS systems Model LYOACC3P1 Cleveland, OH, USA). The drying process was carried out for up to 48 h at −84 °C, with a vacuum at 188 mT. The vials containing the powders were sealed and stored protected from light in a refrigerator for subsequent analyses.

### 2.5. Encapsulation Efficiency

The encapsulation efficiency (EE%) was determined according to Equation (2):

(2) $$\mathrm{EE}\%=\frac{\mathrm{Ce}}{\mathrm{Ci}}\times 100$$

where *EE*% is the encapsulation efficiency, *Ci* is the initial bixin concentration in the suspension, and *Ce* is the bixin concentration in the encapsulated sample.

The bixin concentration in the encapsulated sample was determined according to Equation (3):

*Ce* = *Ci* − *Cs* (3)

where *Cs* is the surface bixin concentration (unencapsulated sample).

The determination of bixin concentrations in the initial (*Ci*) and surface (*Cs*) samples was performed based on the fact that this carotenoid is lipophilic and soluble in hexane and that the matrix (maltodextrin) is, on the other hand, soluble in water and insoluble in hexane. The hexane solvent was chosen because it is non-polar (dipole moment 0.00 D), i.e., bixin crystals are soluble in this solvent.

The surface bixin concentration was determined by washing 50 mg aliquots of the lyophilized powder with 20 mL of hexane in a centrifuge tube for 15 s at 100 rpm. Then, the sample was centrifuged (Hettich Routina 420R, Tuttlingen, Germany) at 528× *g* for 1 min, and the absorbance of the supernatant was measured at 455 nm in a spectrophotometer (Thermoscientific Genesys 10S, Thermo Fisher Scientific, Waltham, MA, USA) [27].

A mass of 50 mg of the lyophilized sample was dispersed in 2.5 mL of water and 20 mL of hexane to set up the initial concentration of bixin. The sample was shaken in a centrifuge (Hettich Routina 420R, Tuttlingen, Germany) at 2500 rpm for 20 min, and aliquots of the organic phase were taken for spectrophotometric analysis at 455 nm.

Bixin quantifications by spectrophotometry were performed at 455 nm, the wavelength of maximum absorption of bixin when solubilized in hexane. A standard curve of absorbance × concentration was plotted for the quantification of bixin in the samples, resulting in a linear calibration curve $(Y=2883.7x+0.0008;\;R^{2}=1).$

### 2.6. Solubility in Water

The water solubility of the microencapsulated powder was evaluated following the methodology described by Eastman and Moore (1984) [28], with adaptations. A mass corresponding to 0.5 g of the sample was diluted in 50 mL of deionized water. Following five minutes of magnetic stirring to homogenize the suspension, the sample was centrifuged for five minutes at 4500 rpm. An aliquot of 12.5 mL of the supernatant was placed in an oven at 105 °C until constant weight, and the solubility (*S*) was calculated, in percentage, by the weight difference, using Equation (4). The analysis was performed in triplicate.

(4) $$S\%=\frac{M_{S}}{M_{t}}\times 100$$

where *M_S_* is the mass of powder in the supernatant and *M_t_* is the total powder mass.

### 2.7. Moisture

The water content of the microencapsulated powder was determined from the mass loss of 5 g of lyophilized sample submitted to heating at 105 °C until constant weight was obtained.

### 2.8. HPLC Analysis

The analysis of the bixin obtained in the extraction step and a sample of standard bixin (Sigma Aldrich Brazil LTDA, St. Louis, MO, USA) with purity ≥ 90% were performed. Samples containing a mass of 0.01 g were prepared and dissolved in the mobile phase methanol/0.5% acetic acid (70:30 *v*/*v*), according to Rahmalia et al. (2015) [29]. The samples were vacuum filtered (Prismatec, Sao Paulo, Brazil) on a Millipore 0.22 µm membrane (Chromafil^®^ Xtra PVDF -20/25, Macherey-Nagel, Düren, Germany) before being injected into the chromatograph. A C18 chromatography column (ShimParck CLC ODS (M), Shimadzu, Kyoto, Japan, 25 cm) was used, maintained at 29 °C, mobile phase under isocratic conditions, and flow rate of 1.0 mL/min. The chromatographic analyses were performed on a Shimadzu chromatograph (CGM model—20A, Columbia, MD, USA). The chromatograms were recorded at a wavelength of 455 nm, with a chromatographic run duration of 10 min, and the spectra were obtained between 250 and 600 nm.

Table 3 shows the chromatogram area and bixin concentration results that were obtained for the injection of volumes of 20, 30, 40, and 50 µL of the bixin sample obtained by extraction. The concentration values were calculated based on the calibration curve of the bixin standard obtained by HPLC ($Y=1306.4x;\;R^{2}=0.0079$), considering the area values within the range of the standard curve (Table 4).

## 3. Results and Discussion

Table 5 shows the approximate composition of bixin seeds. According to Scotter (2009) [4], annatto seeds typically include 1 to 6.3% bixin, 5.4 to 6.9% ash, 9.6 to 13.3% moisture, 2.0 to 4.8% lipids, 12.1 to 17.0% protein, and 70% of total carbs. Similar values were also obtained for annatto seeds in natura variety Piave by Albuquerque and Meireles (2012) [30], namely 4.9% ± 0.2% bixin, 6.2% ± 0.1% ash, 12.3% ± 0.1% moisture, 3.7% ± 0.0% lipids, 12.1% ± 0.2% protein, and 65.7% total carbohydrates.

**Figure 4:**
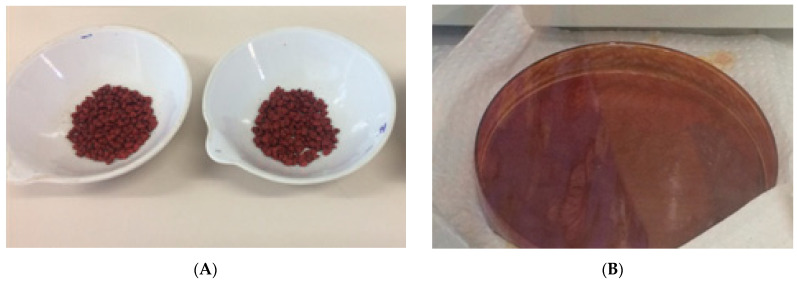
Visual appearance of the annatto seeds in natura (**A**) and the bixin crystals extracted from the seeds (**B**).

For the integer annatto seeds in natura, the following values have been reported: moisture 14.0 ± 0.1%, lipids 4.0 ± 0.3%, bixin 2.0 ± 0.2%, and other material up to 80% [31]. Thus, considering the variability of cultivars, soil, and storage conditions, the values found here differ from the mentioned data. The yield of the bixin extraction process from annatto seeds is a determining factor for the economic viability of this carotenoid in the industry. The extraction of bixin crystals has its yield calculated by the ratio between the amount of powder produced at the end of the extraction and the initial amount of seeds. Using the methodology adopted in this work, a yield of 1.5 ± 0.2% was obtained. Figure 4 shows the visual appearance of the annatto seeds in natura and the bixin crystals extracted from the seeds.

After harvesting and especially during storage, annatto seeds are exposed to some environmental factors, such as water activity, high temperatures, exposure to air, and high relative humidity. Variations in these factors also end up interfering with the yield of pigment production. The content of these compounds present in annatto seeds varies according to the variety of the crop, soil, climate, and cultural practices, and seeds with less than 1% and others with up to 6% of bixin can be found [32]. According to Carvalho et al. (2010) [33], the extraction techniques mostly aim to produce an intense red powder in which bixin, which is most responsible for the color, is in low concentration.

Rahmalia et al. (2014) [9] analyzed the concentration of bixin in annatto seeds using an extraction method with cyclohexane/acetone solvents in different proportions. The authors observed that the amount of carotenoid in their samples was 27.90 ± 0.02 mg bixin/g (2.79 ± 0.02% by weight). The average bixin concentration was reported to range from 12 to 23 mg/g in the seeds, depending on the climatic conditions of planting (such as temperature, lighting, rainfall, and soil) and genetic factors (cultivars).

Chisté et al. (2011) [34] employed intact annatto seeds with water, ethanol, ethyl acetate, ethanol/water (1:1, *v*/*v*), and ethanol/ethyl acetate (1:1, *v*/*v*). On an orbital shaker, solvents were introduced at a mass/solvent ratio of 1:2 (*m*/*v*). The highest bixin content was found in the extracts made with ethanol, ethyl acetate, and ethanol/ethyl acetate. Bixin has the property of being lipid soluble and, therefore, subject to extraction with several organic solvents, among which the most used are chloroform, acetone, propylene glycol, and ethanol [9].

Factors such as choice of solvent, proportion between solvents, extraction temperature, and the amount of water present in the grains influence the final amount of bixin obtained [35].

The difficulties in comparing the results of bixin content from different origins and varieties may be related to the lack of uniformity in the methods of analysis. The methodologies used range from the use of different solutions for extracting the pigments to the inappropriate use of absorption coefficients for quantification [33].

**Figure 5:**
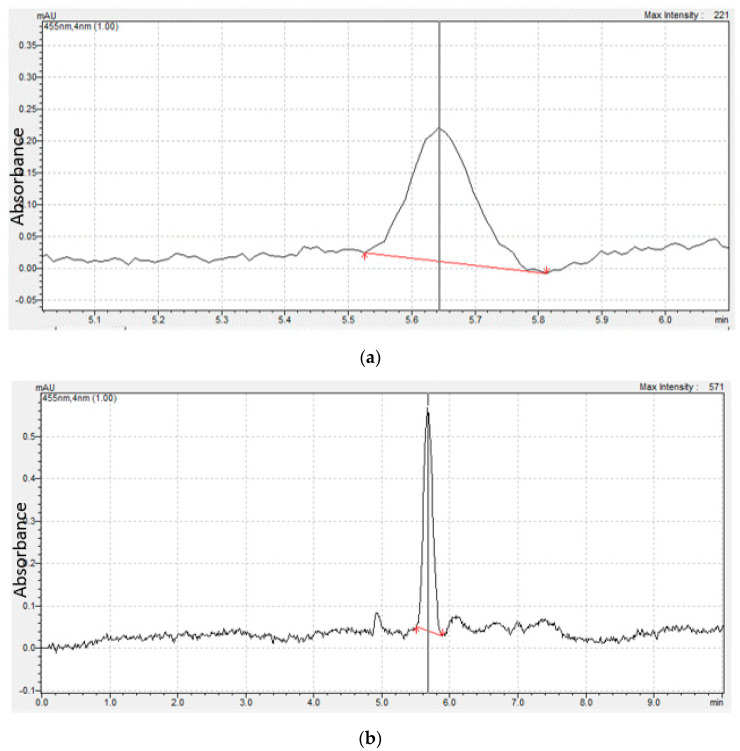
Chromatogram of the bixin standard (>90% purity) (**a**) and the bixin sample obtained after extraction (**b**).

Figure 5 shows the chromatogram of the bixin standard (>90% purity—Figure 5a) and the bixin sample obtained after extraction (Figure 5b).

Figure 5 shows that, under the experimental conditions used, the chromatographic separation indicates the appearance of a peak referring to the bixin pattern corresponding to a retention time of about 5.65 s, which overlaps with the peak of the extract obtained from annatto seed (Figure 5b). The results indicated that the bixin concentration in the extracted sample was 0.12 ± 0.01 g/L.

Montenegro et al. (2004) [14] verified the effect of different conditions on the organic extraction of norbixin and bixin, followed by crystallization from extruded snacks. The authors used HPLC with a mobile phase of acetonitrile/2% acetic acid (65:35) and an analysis wavelength of 461 nm. Two significant retention times were found: a Tr between 18 and 22 min, identified as bixin, and a Tr between 6 and 10 min, identified as norbixin.

Chisté et al. (2011) [34], using an extract with 98% purity, identified bixin as the peak that appears at 38.6 min. Cardeñosa et al. (2011) [36] evaluated the concentration of both pigments (norbixin and bixin) in foods and observed retention times between 8 and 10 min for bixin and between 4 and 6 min for norbixin.

As observed, the values found in the literature for the Tr of bixin in the HPLC analysis depend on factors such as extraction methodology, type of solvent used as the mobile phase, and the proportion of solvents between the stationary phase and the mobile phase. Thus, the results found in this work (annatto extract and bixin standard samples with retention times between 5 and 6 min) indicated that the methodology proposed for the quantification of bixin was successful, confirming that the crystals obtained from the annatto seed extraction procedure were formed by bixin.

**Figure 6:**
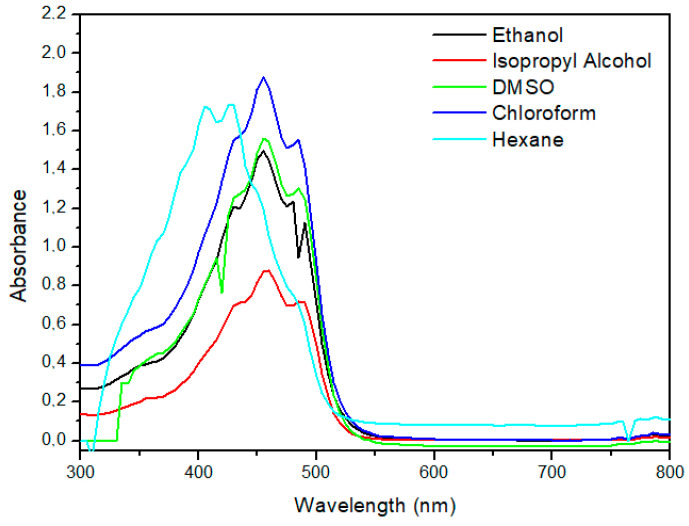
Scanning spectrophotometry of bixin extracted from annatto seeds in the solvents (ethanol, isopropyl alcohol, DMSO, chloroform, and hexane), recorded in the measurement wavelength range from 300 to 800 nm.

The scan results are shown in Figure 6.

Because of their chemical structure, the majority of carotenoids exhibit an absorption peak in the visible region of the spectrum, i.e., between 400 and 500 nm, determined by the long conjugated double bond system of these compounds. According to Generalić Mekinić et al. (2023) [37], the spectrophotometric profile of carotenoids, such as bixin, has three absorption peaks, the last two of which have greater intensity. As seen in Figure 6, the profile is repeated for bixin in all tested solvents. The author reports that the spectra can be used to identify carotenoids since each molecule has specific peaks of wavelengths.

According to Rahmalia et al. (2014) [9], above 400 nm, bixin’s conjugated double-bond structure produces a prominent absorption band and the UV–visible spectra of bixin present a pronounced dependence on the solvent. The solvents that showed the highest absorptions were chloroform and hexane, with wavelength values of 455 and 430 nm, respectively.

Bixin is a fat-soluble organic compound, soluble in alkaline solutions and insoluble in water [10,11]. Research on trans carotenoids, including β-carotene, zeaxanthin, and canthaxanthin, suggests that the trans geometry has the lowest energy structure and is thought to be water-insoluble [38].

Since the chromophore group makes up a significant amount of the molecule, the location of the absorption peak in spectrophotometry provides structural information about the complex. The intensity of the absorption peak is correlated with the carotenoid’s content as well as its structure. In addition, the position and intensity of the absorption peak are sensitive to environmental variations, such as solvent polarity [37].

Annatto carotenoids, mainly bixin, are found in nature, mostly in the cis conformation [4] and even so they present themselves as low polarity compounds and, therefore, soluble in organic solvents. According to Tay-Agbozo et al. (2018) [39], this is brought about by the existence of weak, reversible interactions such as dipole forces, van der Waals interactions, and hydrogen bonds [40], showing a hydrophobic effect.

Considering the adopted methodology, the lipid-soluble condition of bixin, and the literature data, it is observed that the carotenoid concentration in the organic extract will depend on its interaction with the chosen solvent, varying as the solute/solvent ratio is established.

**Figure 7:**
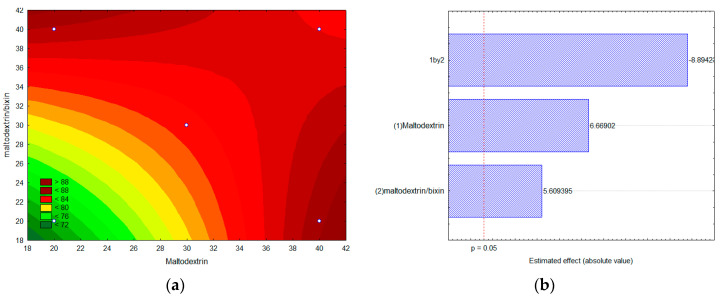
Contour curves (**a**) and Pareto diagram (**b**) for the microencapsulation efficiency response variable as a function of the variables (maltodextrin (1) concentration and maltodextrin/bixin (2) ratio).

Figure 7 shows the contour curves (Figure 7a) and Pareto diagram (Figure 7b) for the microencapsulation efficiency response variable as a function of the variable’s maltodextrin concentration (Figure 7b, column 1) and maltodextrin/bixin ratio (Figure 7b, column 2).

The results show that for the encapsulation efficiency response variable, the maltodextrin concentration and maltodextrin/bixin ratio variables, as well as the interaction between them, were significant at the 95% confidence level (*p* > 0.5). The highest encapsulation efficiencies were obtained for higher concentrations of maltodextrin and for higher values of the maltodextrin/bixin ratio. The lowest value obtained for the encapsulation efficiency (EE%) was with 20% of maltodextrin and maltodextrin/bixin ratio of 20 (72.5%), while the highest value of EE% (85.5%) was observed for a maltodextrin concentration of 40% at the same maltodextrin/bixin ratio.

Curi-Borda et al. (2019) [41] used maltodextrin as a wall material in combination with other compounds to encapsulate annatto dyes. The authors reported that when maltodextrin was used alone, microcapsules were formed with an encapsulation efficiency of 77%. However, when it was associated with a native carbohydrate fraction (gum Arabic, carboxymethylcellulose, and pectin), an increase in efficiency was observed for values above 90%.

It is well recognized that the kind of formulation and method employed can have an impact on the amount of a drug that can be included in micro-encapsulated systems [42], which explains the different efficiency values published in the literature. In this work, maltodextrin was shown to be a suitable material to obtain a high encapsulation efficiency (above 70%) under all investigated experimental conditions. Studies that used the spray drying encapsulation methodology for bixin [43], carotenoids [44], and lycopene [45] reported efficiency values of 86.0%, 82.2%, and 87.5%, respectively.

The type of formulation and employed method determine how much of a drug can be included in encapsulated systems [42]. For Pérez-Monterroza (2018) [46], using the alkaline technique and ultrasound methods, the encapsulation effectiveness of bixin in starch ranged from 17.3% to 94.5% and from 13.1% to 62.1%, respectively.

**Figure 8:**
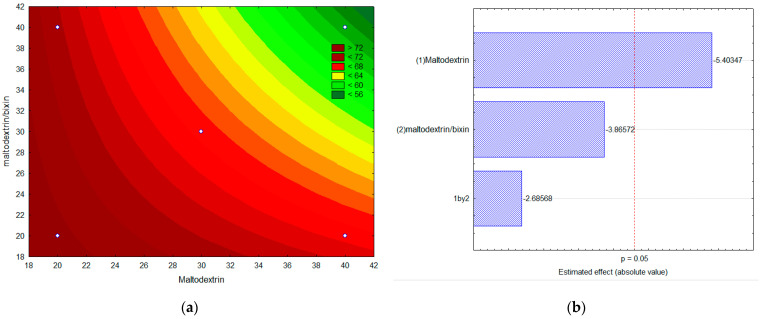
Contour curves (**a**) and Pareto diagram (**b**) for the variable response solubility of the microencapsulated powder as a function of the variables’ maltodextrin (1) concentration and maltodextrin/bixin (2) ratio.

Figure 8 shows the contour curves (Figure 8a) and Pareto diagram (Figure 8b) for the variable solubility response of the microencapsulated powder in water as a function of the variables’ maltodextrin concentration (Figure 8b, column 1) and maltodextrin/bixin ratio (Figure 8b, column 2).

It is observed that only the variable maltodextrin concentration was significant (*p* > 0.5) but with a negative effect (−5.40), i.e., the higher the maltodextrin concentration, the lower the solubility of the microencapsulated powder. Higher solubilities were obtained for lower concentrations of maltodextrin and for lower values of the maltodextrin/bixin ratio.

The highest solubility value in water (75.3%) was observed with maltodextrin 20% *m*/*v* and maltodextrin/bixin ratio of 20, while the lowest solubility (59.5%) was obtained for maltodextrin 40% *m*/*v* and maltodextrin/bixin of 40. Thus, it is observed that the microencapsulation of bixin in maltodextrin favored the solubility of bixin in water. Previous experiments were carried out to assess the degree of solubility of bixin crystals in water, and the results showed that the crystals were insoluble.

The obtained results are similar to those reported by Barbosa et al. (2005) [43]. The authors, when evaluating the solubility of encapsulated and non-encapsulated bixin in water, found that the microcapsules had good solubility (64 s), while the pure crystals were considered insoluble.

Another study that also demonstrated the solubility of the microencapsulated in comparison with the carotenoid crystal was carried out by Santos et al. (2005) [47], who evaluated paprika microcapsules in oleoresin using carbohydrates as wall material. Sousdaleff et al. (2013) [48], when evaluating the solubility of curcumin after lyophilization in maltodextrin, concluded that curcumin microencapsulated at a ratio of 1:20 improved the solubility of the compound after 1 min and 35 s without producing precipitates or agglomerates. This outcome demonstrates that the solubility of this dye is improved when maltodextrin and lyophilization are coupled.

Once the microcapsule wall material dissolves, the hydrophobic substance found in the microcapsule core disperses in water. Therefore, the stability of the aqueous dispersion resulting from the hydrophobic substance will be influenced by multiple factors, including particle size and distribution, ambient circumstances, and the repulsion or attraction between particles [49]. The various instability mechanisms that result from the interaction of these variables include sedimentation, flocculation, and coalescence of hydrophobic particles [41], which could account for the variations in solubilities observed in the tests conducted in our study.

There are many different ways to microencapsulate; therefore, selecting a drying process is essential to obtain the desired result. A number of considerations need to be made, including the distinct chemical and physical characteristics of the encapsulated drug and the core material, their non-reactivity, long-term stability, and potential applications. The size of the capsule and its shape are also crucial.

No drying technique exists that enables carotenoid stability without requiring the addition of wall material. For instance, the creation of viscous and sticky particles happens when a product is dried using a spray dryer without the addition of an encapsulating agent, which is detrimental to the product’s subsequent application. Low molecular weight compounds, which have a low glass transition temperature, are the cause of this [50]. The problem of increasing the temperature of the glassy state is solved by using encapsulating agents, which, when added to the suspension, also influence the final properties of the product. The results of the experimental design indicated that the encapsulation efficiency increases for higher concentrations of maltodextrin. One hypothesis is that the increase in maltodextrin concentration changes the glass transition temperature of the sample during the encapsulation process, favoring the formation of microcapsules.

The average moisture content of the freeze-dried powders varied between 4.1% and 6.1% (standard deviation of 0.64%). According to Tupuna et al. (2018) [51], factors that affect the moisture content include the drying temperature, the proportion of total soluble solids in each sample, and the wall material’s particular hygroscopicity.

Considering that the operational drying parameters were kept constant for all experiments in this study, the variations in the moisture percentages of the microencapsulated powders are due to the characteristics of each sample (maltodextrin concentration and different maltodextrin/bixin ratios). The amount of moisture in powders can have a big impact on their quality because low humidity ensures stability under storage.

Because it can produce yellow to red-orange pigmentation depending on application and dose rate, the naturally occurring pigment bixin is frequently utilized in the food sector. Oil-based food applications, such as dairy spreads, cheese sauces, and processed cheese, are the main uses for bixin due to its oil solubility. To improve its processing in foods and enhance its anti-inflammatory and antioxidant properties [52], encapsulation techniques are found useful for the development of functional foods. Superoxide, hydroxyl, and peroxyl radicals are examples of reactive oxygen and nitrogen species that they can scavenge. By preventing lipid oxidation through the use of bixin as a natural antioxidant, the preservation of foods and other lipid-based ingredients can be extended [53].

## 4. Conclusions

Carotenoids are unstable natural pigments that need protection. Among the numerous techniques that can be used for the microencapsulation of carotenoids, lyophilization stands out. The technique of microencapsulation by lyophilization of bixin using maltodextrin as wall polymer allowed the production of microcapsules with high encapsulation efficiency depending on the maltodextrin concentration in the initial suspension and the Me/Mb ratio, with higher values of encapsulation efficiency being observed for higher maltodextrin concentrations and higher values of the Me/Mb ratio. Regarding the solubility of microencapsulated bixin, the highest solubilities were obtained for lower maltodextrin concentrations and for lower values of the maltodextrin/bixin ratio, having only the maltodextrin concentration with a significant difference (*p* < 0.05). In this way, it can be said that the drying by lyophilization was successful, generating powders that are easy to solubilize in water, which does not happen with the non-encapsulated active material since it is hydrophobic. Changing the solubility of the material without losing its properties is of great importance for the food industry, being an alternative to protect these compounds from factors that cause the oxidation and degradation of bixin, thus improving its stability. Future research will focus on developing green extraction techniques for bixin that are aligned with sustainable procedures and minimizing environmental impact. Although traditional solvent-based methods are effective, adopting ecological techniques could significantly expand the applicability of bixin in the food and pharmaceutical industries. The goal is to maintain extraction efficacy and quality while promoting the use of bixin in food and pharmaceutical applications. This approach will not only address environmental concerns but also enhance the commercial value of bixin in an increasingly sustainability-oriented market. Additionally, research will be deepened on encapsulation methods, aiming to increase the solubility and stability of the extracted compounds, which is crucial for improving the functionality and effectiveness of bixin in various applications.

## Figures and Tables

**Table 1:** Variables investigated in the experimental design.

Variable	−1	0	+1
Maltodextrin concentration (%)	20	30	40
Bixin/maltodextrin ratio (Mb/Me) *	1:20	1:30	1:40

* Mb—bixin mass; Me—encapsulating mass (maltodextrin).

**Table 2:** Central composite planning matrix 2^2^ with five central points (level 0).

Essay	Maltodextrin Concentration (%)	Bixin/Maltodextrin Ratio (Mb/Me) *
1	−1	−1
2	−1	+1
3	+1	−1
4	+1	+1
5	0	0
6	0	0
7	0	0
8	0	0
9	0	0

* Mb—bixin mass; Me—encapsulating mass (maltodextrin).

**Table 3:** Results of the bixin sample obtained by extraction and quantified using high-performance liquid chromatography.

RT * (min)	Area	Volume (µL)	Concentration (g/L)
6.05	3527.4	20	0.1350
5.67	4224.3	30	0.1078
5.79	6514.8	40	0.1247
5.68	7018.6	50	0.1074
		Average ± s.d.	0.12 ± 0.01

* RT—retention time; s.d.—standard deviation.

**Table 4:** Bixin standard quantified by high-performance liquid chromatography.

Bixin Mass (µg)	Area	RT * (min)
0.00	0.00	0.00
0.64	525.60	5.63
1.28	1281.30	5.63
3.84	4952.60	5.67
5.12	6821.40	5.67
5.76	7570.80	5.69

* RT—retention time.

**Table 5:** Physicochemical characterization of annatto seeds.

Component	Concentration (%) (Mean ± s.d. *)
Ash	9.73 ± 0.45
Proteins	12.93 ± 0.15
Lipids	2.12 ± 0.20
Moisture	10.18 ± 0.68

* s.d.—standard deviation.

## Data Availability

The original contributions presented in the study are included in the article, further inquiries can be directed to the corresponding authors.
